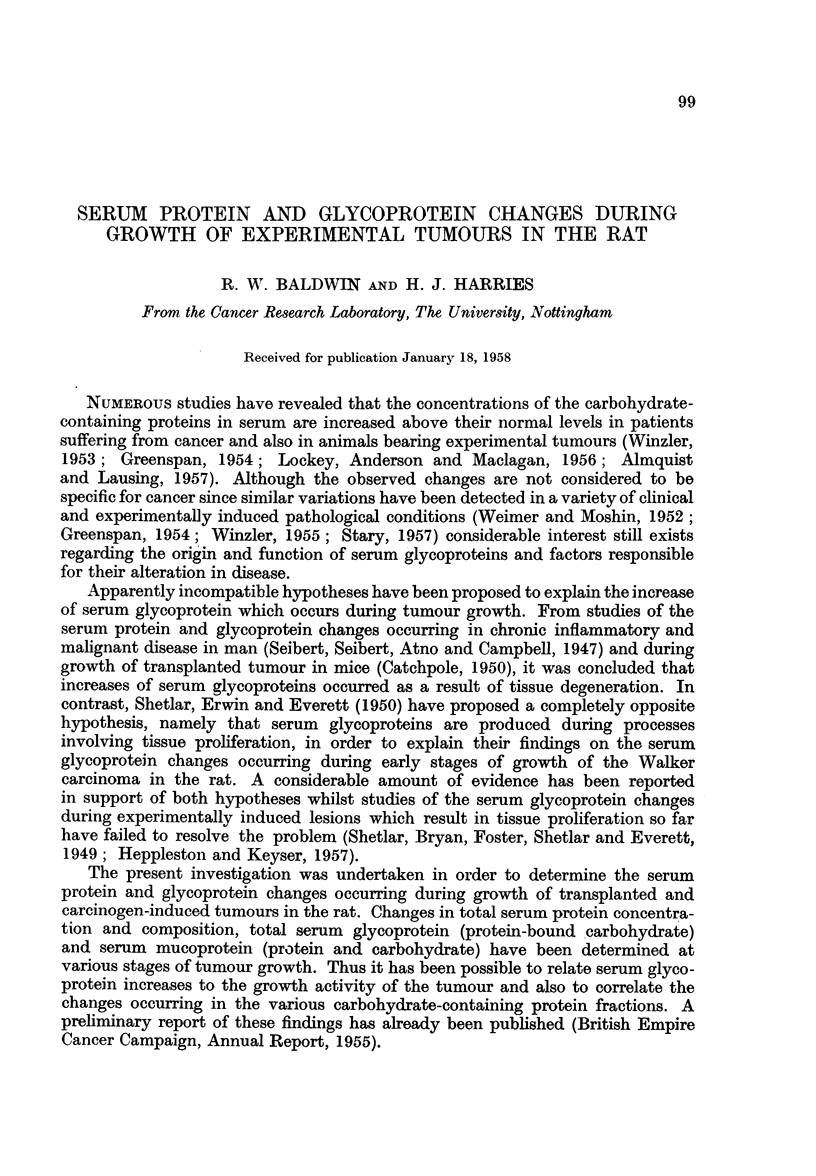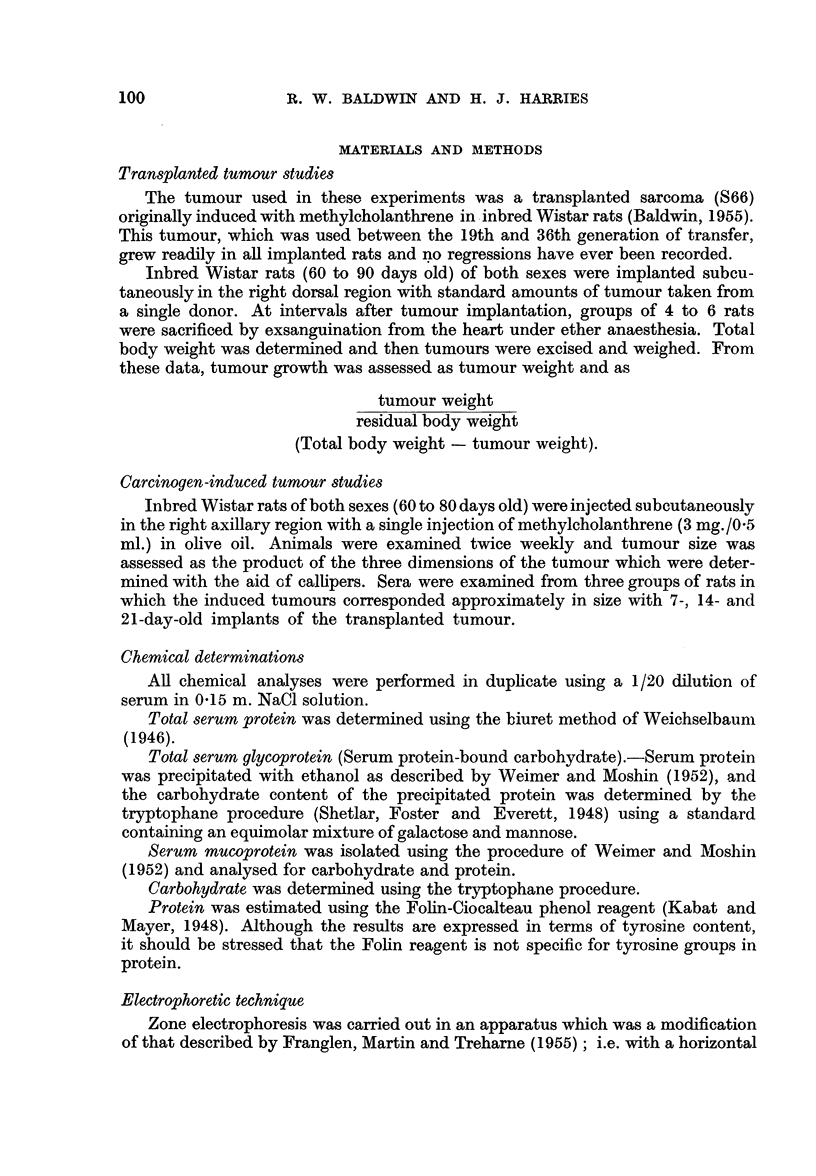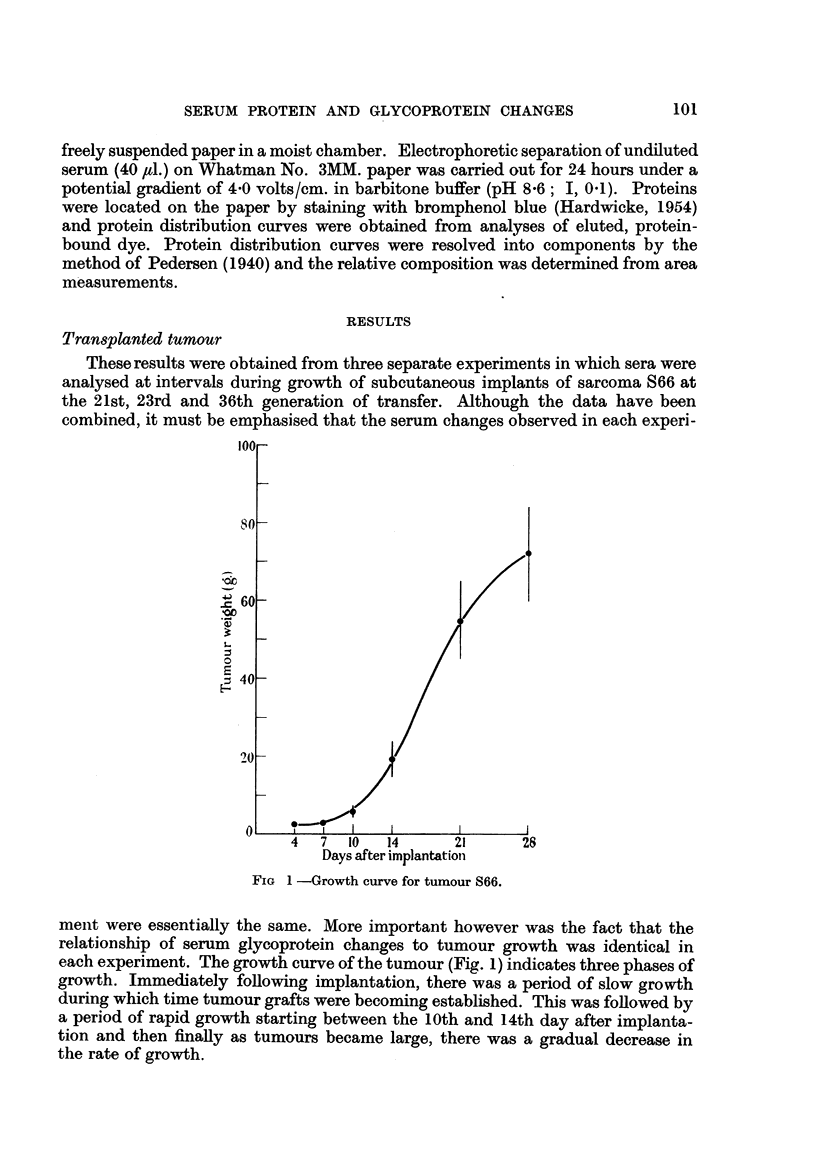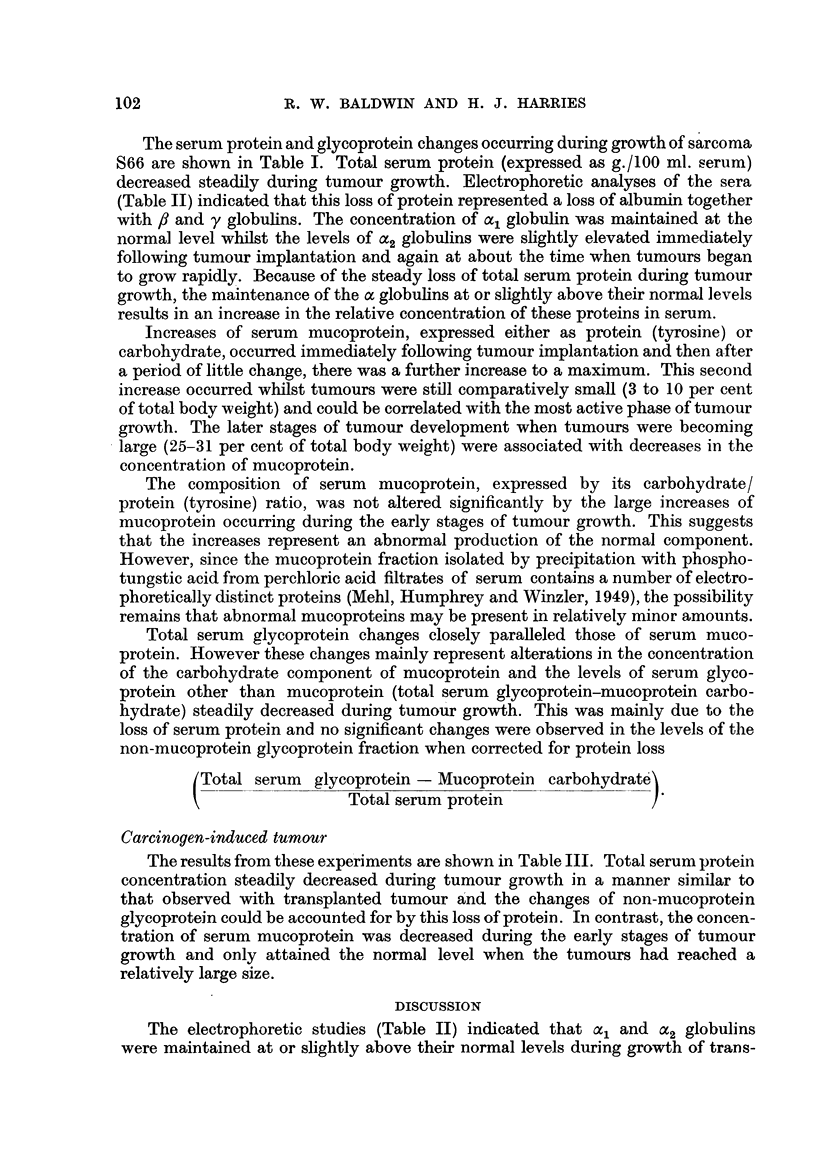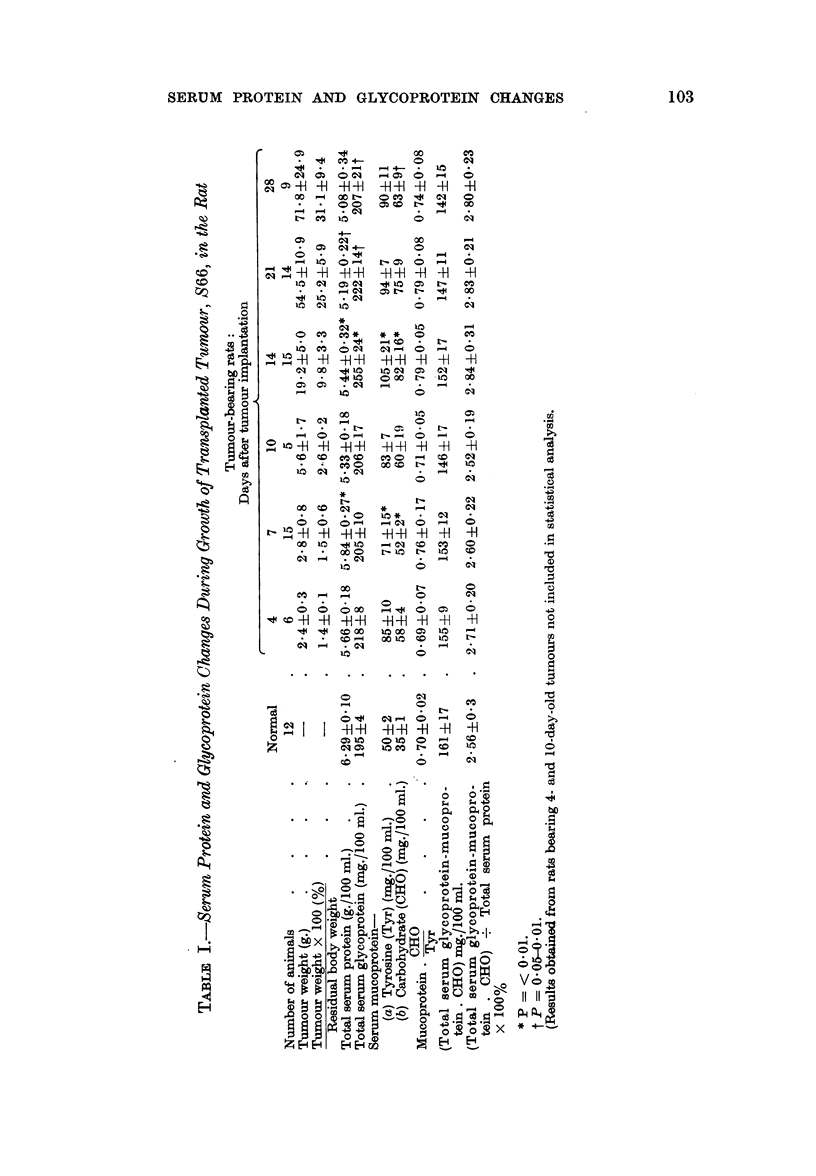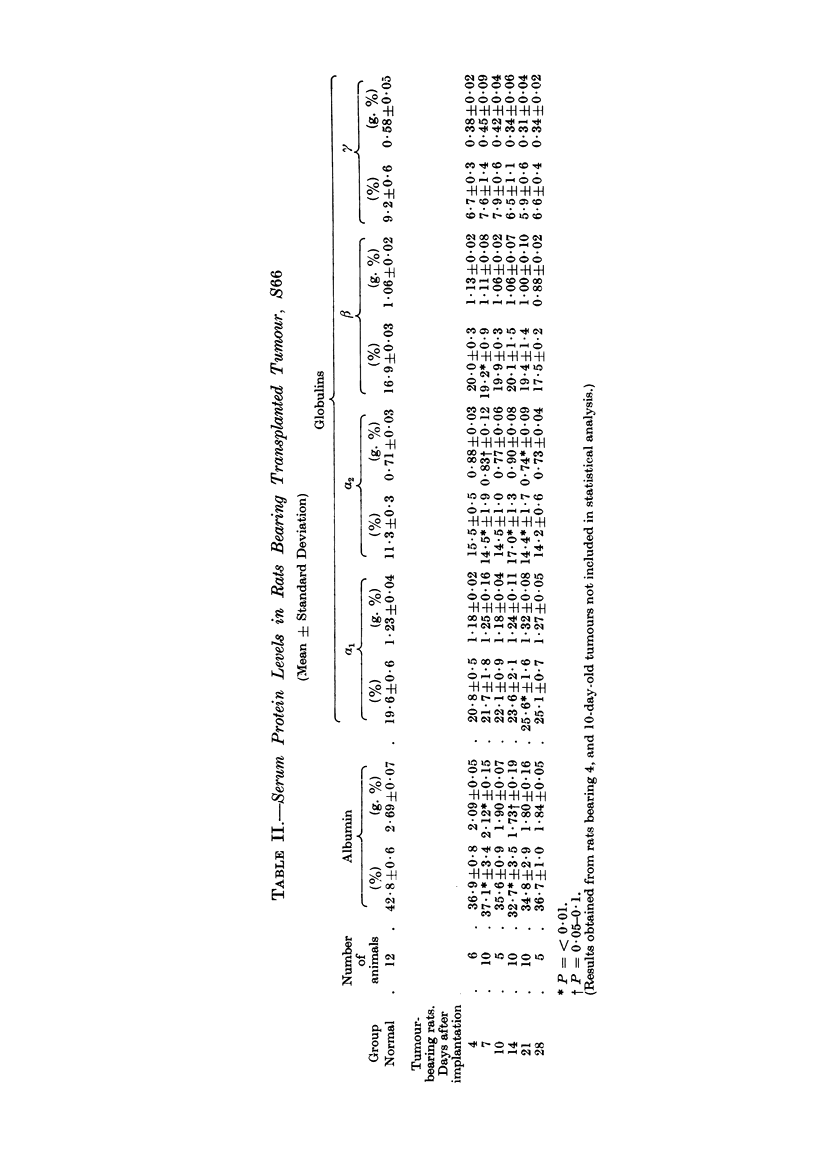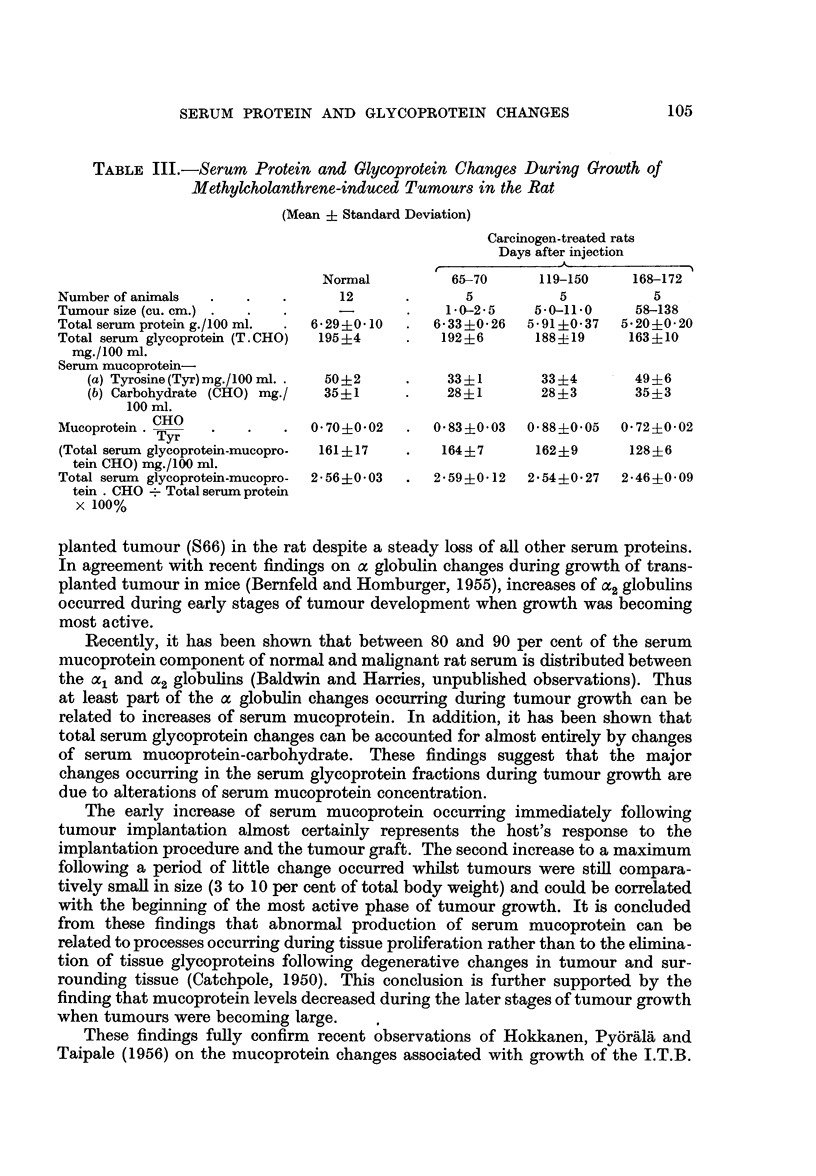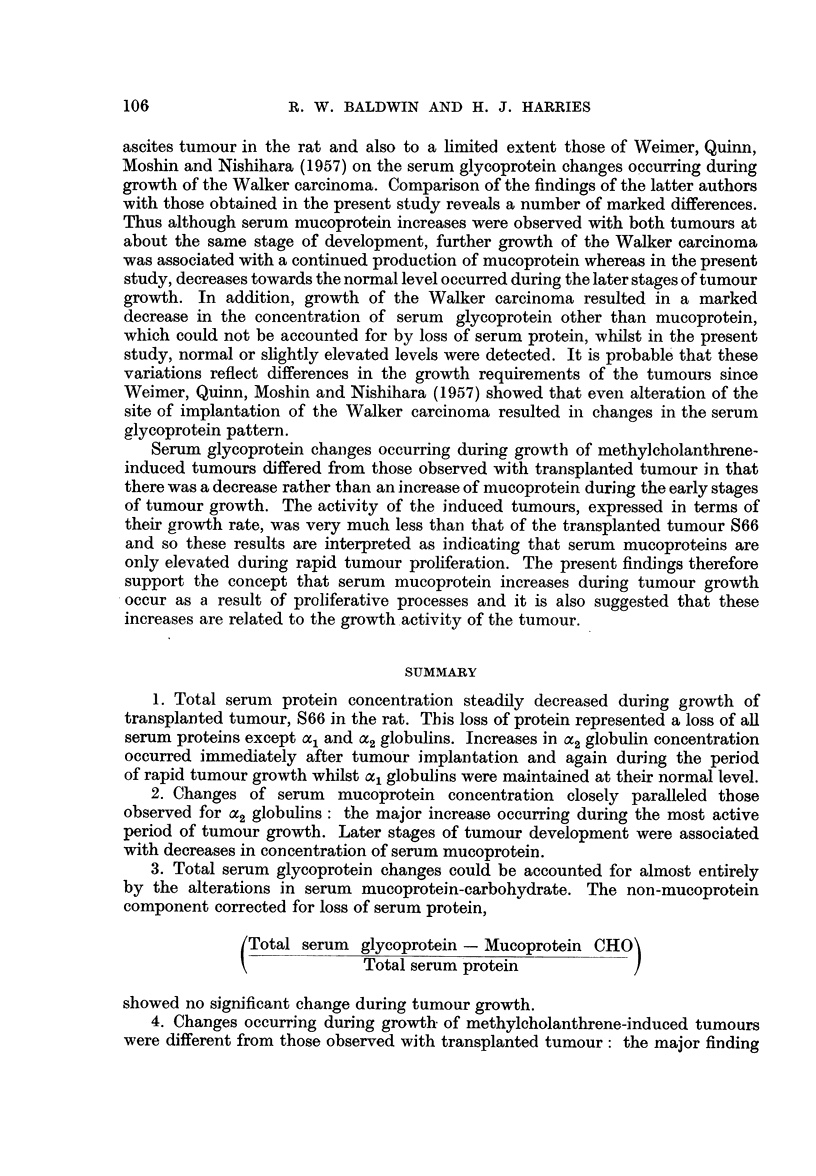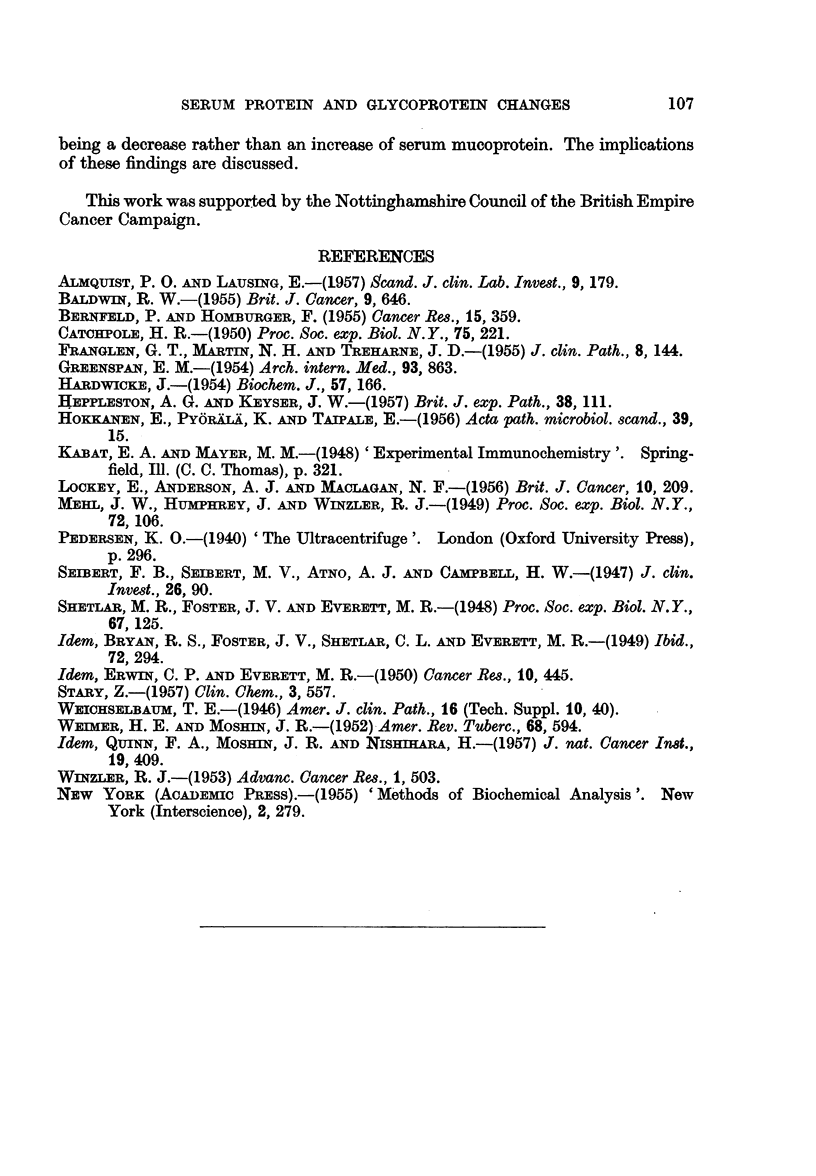# Serum Protein and Glycoprotein Changes During Growth of Experimental Tumours in the Rat

**DOI:** 10.1038/bjc.1958.13

**Published:** 1958-03

**Authors:** R. W. Baldwin, H. J. Harries


### SERUM PROTEIN AND GLYCOPROTEIN CHANGES DURING GROWTH OF EXPERIMENTAL TUMOURS IN THE RAT

R. W. BALDWIN AND H. J. HARRIES

*From the Cancer Research Laboratory, The University, Nottingham*

Received for publication January 18, 1958

NUMEROUS studies have revealed that the concentrations of the carbohydrate-containing proteins in serum are increased above their normal levels in patients suffering from cancer and also in animals bearing experimental tumours (Winzler, 1953; Greenspan, 1954; Lockey, Anderson and Maclagan, 1956; Almquist and Lausing, 1957). Although the observed changes are not considered to be specific for cancer since similar variations have been detected in a variety of clinical and experimentally induced pathological conditions (Weimer and Moshin, 1952; Greenspan, 1954; Winzler, 1955; Stary, 1957) considerable interest still exists regarding the origin and function of serum glycoproteins and factors responsible for their alteration in disease.

Apparently incompatible hypotheses have been proposed to explain the increase of serum glycoprotein which occurs during tumour growth. From studies of the serum protein and glycoprotein changes occurring in chronic inflammatory and malignant disease in man (Seibert, Seibert, Atno and Campbell, 1947) and during growth of transplanted tumour in mice (Catchpole, 1950), it was concluded that increases of serum glycoproteins occurred as a result of tissue degeneration. In contrast, Shetlar, Erwin and Everett (1950) have proposed a completely opposite hypothesis, namely that serum glycoproteins are produced during processes involving tissue proliferation, in order to explain their findings on the serum glycoprotein changes occurring during early stages of growth of the Walker carcinoma in the rat. A considerable amount of evidence has been reported in support of both hypotheses whilst studies of the serum glycoprotein changes during experimentally induced lesions which result in tissue proliferation so far have failed to resolve the problem (Shetlar, Bryan, Foster, Shetlar and Everett, 1949; Heppleston and Keyser, 1957).

The present investigation was undertaken in order to determine the serum protein and glycoprotein changes occurring during growth of transplanted and carcinogen-induced tumours in the rat. Changes in total serum protein concentration and composition, total serum glycoprotein (protein-bound carbohydrate) and serum mucoprotein (protein and carbohydrate) have been determined at various stages of tumour growth. Thus it has been possible to relate serum glycoprotein increases to the growth activity of the tumour and also to correlate the changes occurring in the various carbohydrate-containing protein fractions. A preliminary report of these findings has already been published (British Empire Cancer Campaign, Annual Report, 1955).

### MATERIALS AND METHODS

*Transplanted tumour studies*

The tumour used in these experiments was a transplanted sarcoma (S66) originally induced with methylcholanthrene in inbred Wistar rats (Baldwin, 1955). This tumour, which was used between the 19th and 36th generation of transfer, grew readily in all implanted rats and no regressions have ever been recorded.

Inbred Wistar rats (60 to 90 days old) of both sexes were implanted subcutaneously in the right dorsal region with standard amounts of tumour taken from a single donor. At intervals after tumour implantation, groups of 4 to 6 rats were sacrificed by exsanguination from the heart under ether anaesthesia. Total body weight was determined and then tumours were excised and weighed. From these data, tumour growth was assessed as tumour weight and as

$$\frac{\text{tumour weight}}{\text{residual body weight}}$$

(Total body weight — tumour weight).

*Carcinogen-induced tumour studies*

Inbred Wistar rats of both sexes (60 to 80 days old) were injected subcutaneously in the right axillary region with a single injection of methylcholanthrene (3 mg./0·5 ml.) in olive oil. Animals were examined twice weekly and tumour size was assessed as the product of the three dimensions of the tumour which were determined with the aid of callipers. Sera were examined from three groups of rats in which the induced tumours corresponded approximately in size with 7-, 14- and 21-day-old implants of the transplanted tumour.

*Chemical determinations*

All chemical analyses were performed in duplicate using a 1/20 dilution of serum in 0·15 m. NaCl solution.

*Total serum protein* was determined using the biuret method of Weichselbaum (1946).

*Total serum glycoprotein* (Serum protein-bound carbohydrate).—Serum protein was precipitated with ethanol as described by Weimer and Moshin (1952), and the carbohydrate content of the precipitated protein was determined by the tryptophane procedure (Shetlar, Foster and Everett, 1948) using a standard containing an equimolar mixture of galactose and mannose.

*Serum mucoprotein* was isolated using the procedure of Weimer and Moshin (1952) and analysed for carbohydrate and protein.

*Carbohydrate* was determined using the tryptophane procedure.

*Protein* was estimated using the Folin-Ciocalteau phenol reagent (Kabat and Mayer, 1948). Although the results are expressed in terms of tyrosine content, it should be stressed that the Folin reagent is not specific for tyrosine groups in protein.

*Electrophoretic technique*

Zone electrophoresis was carried out in an apparatus which was a modification of that described by Franglen, Martin and Treharne (1955) ; i.e. with a horizontal freely suspended paper in a moist chamber. Electrophoretic separation of undiluted serum (40 $\mu$l.) on Whatman No. 3MM. paper was carried out for 24 hours under a potential gradient of 4·0 volts/cm. in barbitone buffer (pH 8·6 ; I, 0·1). Proteins were located on the paper by staining with bromphenol blue (Hardwicke, 1954) and protein distribution curves were obtained from analyses of eluted, protein-bound dye. Protein distribution curves were resolved into components by the method of Pedersen (1940) and the relative composition was determined from area measurements.

### RESULTS

### *Transplanted tumour*

These results were obtained from three separate experiments in which sera were analysed at intervals during growth of subcutaneous implants of sarcoma S66 at the 21st, 23rd and 36th generation of transfer. Although the data have been combined, it must be emphasised that the serum changes observed in each experi-

FIG 1 —Growth curve for tumour S66.

ment were essentially the same. More important however was the fact that the relationship of serum glycoprotein changes to tumour growth was identical in each experiment. The growth curve of the tumour (Fig. 1) indicates three phases of growth. Immediately following implantation, there was a period of slow growth during which time tumour grafts were becoming established. This was followed by a period of rapid growth starting between the 10th and 14th day after implantation and then finally as tumours became large, there was a gradual decrease in the rate of growth.

The serum protein and glycoprotein changes occurring during growth of sarcoma S66 are shown in Table I. Total serum protein (expressed as g./100 ml. serum) decreased steadily during tumour growth. Electrophoretic analyses of the sera (Table II) indicated that this loss of protein represented a loss of albumin together with $\beta$ and $\gamma$ globulins. The concentration of $\alpha_1$ globulin was maintained at the normal level whilst the levels of $\alpha_2$ globulins were slightly elevated immediately following tumour implantation and again at about the time when tumours began to grow rapidly. Because of the steady loss of total serum protein during tumour growth, the maintenance of the $\alpha$ globulins at or slightly above their normal levels results in an increase in the relative concentration of these proteins in serum.

Increases of serum mucoprotein, expressed either as protein (tyrosine) or carbohydrate, occurred immediately following tumour implantation and then after a period of little change, there was a further increase to a maximum. This second increase occurred whilst tumours were still comparatively small (3 to 10 per cent of total body weight) and could be correlated with the most active phase of tumour growth. The later stages of tumour development when tumours were becoming large (25–31 per cent of total body weight) were associated with decreases in the concentration of mucoprotein.

The composition of serum mucoprotein, expressed by its carbohydrate/protein (tyrosine) ratio, was not altered significantly by the large increases of mucoprotein occurring during the early stages of tumour growth. This suggests that the increases represent an abnormal production of the normal component. However, since the mucoprotein fraction isolated by precipitation with phosphotungstic acid from perchloric acid filtrates of serum contains a number of electrophoretically distinct proteins (Mehl, Humphrey and Winzler, 1949), the possibility remains that abnormal mucoproteins may be present in relatively minor amounts.

Total serum glycoprotein changes closely paralleled those of serum mucoprotein. However these changes mainly represent alterations in the concentration of the carbohydrate component of mucoprotein and the levels of serum glycoprotein other than mucoprotein (total serum glycoprotein–mucoprotein carbohydrate) steadily decreased during tumour growth. This was mainly due to the loss of serum protein and no significant changes were observed in the levels of the non-mucoprotein glycoprotein fraction when corrected for protein loss

$$\left(\frac{\text{Total serum glycoprotein} - \text{Mucoprotein carbohydrate}}{\text{Total serum protein}}\right).$$

*Carcinogen-induced tumour*

The results from these experiments are shown in Table III. Total serum protein concentration steadily decreased during tumour growth in a manner similar to that observed with transplanted tumour and the changes of non-mucoprotein glycoprotein could be accounted for by this loss of protein. In contrast, the concentration of serum mucoprotein was decreased during the early stages of tumour growth and only attained the normal level when the tumours had reached a relatively large size.

### DISCUSSION

The electrophoretic studies (Table II) indicated that $\alpha_1$ and $\alpha_2$ globulins were maintained at or slightly above their normal levels during growth of trans-

TABLE I.—*Serum Protein and Glycoprotein Changes During Growth of Transplanted Tumour, S66, in the Rat*

| | Normal | Tumour-bearing rats: Days after tumour implantation 4 | 7 | 10 | 14 | 21 | 28 |
|---|---|---|---|---|---|---|---|
| Number of animals | 12 | 6 | 15 | 5 | 15 | 14 | 9 |
| Tumour weight (g.) | — | 2·4±0·3 | 2·8±0·8 | 5·6±1·7 | 19·2±5·0 | 54·5±10·9 | 71·8±24·9 |
| $\frac{\text{Tumour weight}}{\text{Residual body weight}} \times 100$ (%) | — | 1·4±0·1 | 1·5±0·6 | 2·6±0·2 | 9·8±3·3 | 25·2±5·9 | 31·1±9·4 |
| Total serum protein (g./100 ml.) | 6·29±0·10 | 5·66±0·18 | 5·84±0·27* | 5·33±0·18 | 5·44±0·32* | 5·19±0·22† | 5·08±0·34 |
| Total serum glycoprotein (mg./100 ml.) | 195±4 | 218±8 | 205±10 | 206±17 | 255±24* | 222±14† | 207±21† |
| Serum mucoprotein— | | | | | | | |
| (*a*) Tyrosine (Tyr) (mg./100 ml.) | 50±2 | 85±10 | 71±15* | 83±7 | 105±21* | 94±7 | 90±11 |
| (*b*) Carbohydrate (CHO) (mg./100 ml.) | 35±1 | 58±4 | 52±2* | 60±19 | 82±16* | 75±9 | 63±9† |
| Mucoprotein . $\frac{\text{CHO}}{\text{Tyr}}$ | 0·70±0·02 | 0·69±0·07 | 0·76±0·17 | 0·71±0·05 | 0·79±0·05 | 0·79±0·08 | 0·74±0·08 |
| (Total serum glycoprotein-mucoprotein . CHO) mg./100 ml. | 161±17 | 155±9 | 153±12 | 146±17 | 152±17 | 147±11 | 142±15 |
| (Total serum glycoprotein-mucoprotein . CHO) ÷ Total serum protein × 100% | 2·56±0·3 | 2·71±0·20 | 2·60±0·22 | 2·52±0·19 | 2·84±0·31 | 2·83±0·21 | 2·80±0·23 |

* P = < 0·01.

† *P* = 0·05–0·01.

(Results obtained from rats bearing 4- and 10-day-old tumours not included in statistical analysis.

TABLE II.—*Serum Protein Levels in Rats Bearing Transplanted Tumour, S66*

(Mean ± Standard Deviation)

| Group | Number of animals | Albumin (%) | Albumin (g. %) | Globulins $\alpha_1$ (%) | Globulins $\alpha_1$ (g. %) | Globulins $\alpha_2$ (%) | Globulins $\alpha_2$ (g. %) | Globulins $\beta$ (%) | Globulins $\beta$ (g. %) | Globulins $\gamma$ (%) | Globulins $\gamma$ (g. %) |
|---|---|---|---|---|---|---|---|---|---|---|---|
| Normal | 12 | 42·8±0·6 | 2·69±0·07 | 19·6±0·6 | 1·23±0·04 | 11·3±0·3 | 0·71±0·03 | 16·9±0·03 | 1·06±0·02 | 9·2±0·6 | 0·58±0·05 |
| Tumour-bearing rats. Days after implantation | | | | | | | | | | | |
| 4 | 6 | 36·9±0·8 | 2·09±0·05 | 20·8±0·5 | 1·18±0·02 | 15·5±0·5 | 0·88±0·03 | 20·0±0·3 | 1·13±0·02 | 6·7±0·3 | 0·38±0·02 |
| 7 | 10 | 37·1*±3·4 | 2·12*±0·15 | 21·7±1·8 | 1·25±0·16 | 14·5*±1·9 | 0·83†±0·12 | 19·2*±0·9 | 1·11±0·08 | 7·6±1·4 | 0·45±0·09 |
| 10 | 5 | 35·6±0·9 | 1·90±0·07 | 22·1±0·9 | 1·18±0·04 | 14·5±1·0 | 0·77±0·06 | 19·9±0·3 | 1·06±0·02 | 7·9±0·6 | 0·42±0·04 |
| 14 | 10 | 32·7*±3·5 | 1·73†±0·19 | 23·6±2·1 | 1·24±0·11 | 17·0*±1·3 | 0·90±0·08 | 20·1±1·5 | 1·06±0·07 | 6·5±1·1 | 0·34±0·06 |
| 21 | 10 | 34·8±2·9 | 1·80±0·16 | 25·6*±1·6 | 1·32±0·08 | 14·4*±1·7 | 0·74*±0·09 | 19·4±1·4 | 1·00±0·10 | 5·9±0·6 | 0·31±0·04 |
| 28 | 5 | 36·7±1·0 | 1·84±0·05 | 25·1±0·7 | 1·27±0·05 | 14·2±0·6 | 0·73±0·04 | 17·5±0·2 | 0·88±0·02 | 6·6±0·4 | 0·34±0·02 |

* $P = < 0·01$.

† $P = 0·05–0·1$.

(Results obtained from rats bearing 4, and 10-day-old tumours not included in statistical analysis.)

TABLE III.—*Serum Protein and Glycoprotein Changes During Growth of Methylcholanthrene-induced Tumours in the Rat*

(Mean $\pm$ Standard Deviation)

| | Normal | Carcinogen-treated rats Days after injection 65–70 | 119–150 | 168–172 |
|---|---|---|---|---|
| Number of animals | 12 | 5 | 5 | 5 |
| Tumour size (cu. cm.) | — | 1·0–2·5 | 5·0–11·0 | 58–138 |
| Total serum protein g./100 ml. | 6·29$\pm$0·10 | 6·33$\pm$0·26 | 5·91$\pm$0·37 | 5·20$\pm$0·20 |
| Total serum glycoprotein (T.CHO) mg./100 ml. | 195$\pm$4 | 192$\pm$6 | 188$\pm$19 | 163$\pm$10 |
| Serum mucoprotein— | | | | |
| (*a*) Tyrosine (Tyr) mg./100 ml. | 50$\pm$2 | 33$\pm$1 | 33$\pm$4 | 49$\pm$6 |
| (*b*) Carbohydrate (CHO) mg./100 ml. | 35$\pm$1 | 28$\pm$1 | 28$\pm$3 | 35$\pm$3 |
| Mucoprotein . $\frac{CHO}{Tyr}$ | 0·70$\pm$0·02 | 0·83$\pm$0·03 | 0·88$\pm$0·05 | 0·72$\pm$0·02 |
| (Total serum glycoprotein-mucoprotein CHO) mg./100 ml. | 161$\pm$17 | 164$\pm$7 | 162$\pm$9 | 128$\pm$6 |
| Total serum glycoprotein-mucoprotein . CHO $\div$ Total serum protein $\times$ 100% | 2·56$\pm$0·03 | 2·59$\pm$0·12 | 2·54$\pm$0·27 | 2·46$\pm$0·09 |

planted tumour (S66) in the rat despite a steady loss of all other serum proteins. In agreement with recent findings on $\alpha$ globulin changes during growth of transplanted tumour in mice (Bernfeld and Homburger, 1955), increases of $\alpha_2$ globulins occurred during early stages of tumour development when growth was becoming most active.

Recently, it has been shown that between 80 and 90 per cent of the serum mucoprotein component of normal and malignant rat serum is distributed between the $\alpha_1$ and $\alpha_2$ globulins (Baldwin and Harries, unpublished observations). Thus at least part of the $\alpha$ globulin changes occurring during tumour growth can be related to increases of serum mucoprotein. In addition, it has been shown that total serum glycoprotein changes can be accounted for almost entirely by changes of serum mucoprotein-carbohydrate. These findings suggest that the major changes occurring in the serum glycoprotein fractions during tumour growth are due to alterations of serum mucoprotein concentration.

The early increase of serum mucoprotein occurring immediately following tumour implantation almost certainly represents the host's response to the implantation procedure and the tumour graft. The second increase to a maximum following a period of little change occurred whilst tumours were still comparatively small in size (3 to 10 per cent of total body weight) and could be correlated with the beginning of the most active phase of tumour growth. It is concluded from these findings that abnormal production of serum mucoprotein can be related to processes occurring during tissue proliferation rather than to the elimination of tissue glycoproteins following degenerative changes in tumour and surrounding tissue (Catchpole, 1950). This conclusion is further supported by the finding that mucoprotein levels decreased during the later stages of tumour growth when tumours were becoming large.

These findings fully confirm recent observations of Hokkanen, Pyörälä and Taipale (1956) on the mucoprotein changes associated with growth of the I.T.B.

ascites tumour in the rat and also to a limited extent those of Weimer, Quinn, Moshin and Nishihara (1957) on the serum glycoprotein changes occurring during growth of the Walker carcinoma. Comparison of the findings of the latter authors with those obtained in the present study reveals a number of marked differences. Thus although serum mucoprotein increases were observed with both tumours at about the same stage of development, further growth of the Walker carcinoma was associated with a continued production of mucoprotein whereas in the present study, decreases towards the normal level occurred during the later stages of tumour growth. In addition, growth of the Walker carcinoma resulted in a marked decrease in the concentration of serum glycoprotein other than mucoprotein, which could not be accounted for by loss of serum protein, whilst in the present study, normal or slightly elevated levels were detected. It is probable that these variations reflect differences in the growth requirements of the tumours since Weimer, Quinn, Moshin and Nishihara (1957) showed that even alteration of the site of implantation of the Walker carcinoma resulted in changes in the serum glycoprotein pattern.

Serum glycoprotein changes occurring during growth of methylcholanthrene-induced tumours differed from those observed with transplanted tumour in that there was a decrease rather than an increase of mucoprotein during the early stages of tumour growth. The activity of the induced tumours, expressed in terms of their growth rate, was very much less than that of the transplanted tumour S66 and so these results are interpreted as indicating that serum mucoproteins are only elevated during rapid tumour proliferation. The present findings therefore support the concept that serum mucoprotein increases during tumour growth occur as a result of proliferative processes and it is also suggested that these increases are related to the growth activity of the tumour.

### SUMMARY

1. Total serum protein concentration steadily decreased during growth of transplanted tumour, S66 in the rat. This loss of protein represented a loss of all serum proteins except $\alpha_1$ and $\alpha_2$ globulins. Increases in $\alpha_2$ globulin concentration occurred immediately after tumour implantation and again during the period of rapid tumour growth whilst $\alpha_1$ globulins were maintained at their normal level.

2. Changes of serum mucoprotein concentration closely paralleled those observed for $\alpha_2$ globulins : the major increase occurring during the most active period of tumour growth. Later stages of tumour development were associated with decreases in concentration of serum mucoprotein.

3. Total serum glycoprotein changes could be accounted for almost entirely by the alterations in serum mucoprotein-carbohydrate. The non-mucoprotein component corrected for loss of serum protein,

$$\left(\frac{\text{Total serum glycoprotein} - \text{Mucoprotein CHO}}{\text{Total serum protein}}\right)$$

showed no significant change during tumour growth.

4. Changes occurring during growth of methylcholanthrene-induced tumours were different from those observed with transplanted tumour : the major finding being a decrease rather than an increase of serum mucoprotein. The implications of these findings are discussed.

This work was supported by the Nottinghamshire Council of the British Empire Cancer Campaign.

### REFERENCES

ALMQUIST, P. O. AND LAUSING, E.—(1957) *Scand. J. clin. Lab. Invest.*, **9**, 179.
BALDWIN, R. W.—(1955) *Brit. J. Cancer*, **9**, 646.
BERNFELD, P. AND HOMBURGER, F. (1955) *Cancer Res.*, **15**, 359.
CATCHPOLE, H. R.—(1950) *Proc. Soc. exp. Biol. N.Y.*, **75**, 221.
FRANGLEN, G. T., MARTIN, N. H. AND TREHARNE, J. D.—(1955) *J. clin. Path.*, **8**, 144.
GREENSPAN, E. M.—(1954) *Arch. intern. Med.*, **93**, 863.
HARDWICKE, J.—(1954) *Biochem. J.*, **57**, 166.
HEPPLESTON, A. G. AND KEYSER, J. W.—(1957) *Brit. J. exp. Path.*, **38**, 111.
HOKKANEN, E., PYÖRÄLÄ, K. AND TAIPALE, E.—(1956) *Acta path. microbiol. scand.*, **39**, 15.
KABAT, E. A. AND MAYER, M. M.—(1948) 'Experimental Immunochemistry'. Springfield, Ill. (C. C. Thomas), p. 321.
LOCKEY, E., ANDERSON, A. J. AND MACLAGAN, N. F.—(1956) *Brit. J. Cancer*, **10**, 209.
MEHL, J. W., HUMPHREY, J. AND WINZLER, R. J.—(1949) *Proc. Soc. exp. Biol. N.Y.*, **72**, 106.
PEDERSEN, K. O.—(1940) 'The Ultracentrifuge'. London (Oxford University Press), p. 296.
SEIBERT, F. B., SEIBERT, M. V., ATNO, A. J. AND CAMPBELL, H. W.—(1947) *J. clin. Invest.*, **26**, 90.
SHETLAR, M. R., FOSTER, J. V. AND EVERETT, M. R.—(1948) *Proc. Soc. exp. Biol. N.Y.*, **67**, 125.
*Idem*, BRYAN, R. S., FOSTER, J. V., SHETLAR, C. L. AND EVERETT, M. R.—(1949) *Ibid.*, **72**, 294.
*Idem*, ERWIN, C. P. AND EVERETT, M. R.—(1950) *Cancer Res.*, **10**, 445.
STARY, Z.—(1957) *Clin. Chem.*, **3**, 557.
WEICHSELBAUM, T. E.—(1946) *Amer. J. clin. Path.*, **16** (Tech. Suppl. **10**, 40).
WEIMER, H. E. AND MOSHIN, J. R.—(1952) *Amer. Rev. Tuberc.*, **68**, 594.
*Idem*, QUINN, F. A., MOSHIN, J. R. AND NISHIHARA, H.—(1957) *J. nat. Cancer Inst.*, **19**, 409.
WINZLER, R. J.—(1953) *Advanc. Cancer Res.*, **1**, 503.
NEW YORK (ACADEMIC PRESS).—(1955) 'Methods of Biochemical Analysis'. New York (Interscience), **2**, 279.